# Activation of GPER-1 Estradiol Receptor Downregulates Production of Testosterone in Isolated Rat Leydig Cells and Adult Human Testis

**DOI:** 10.1371/journal.pone.0092425

**Published:** 2014-04-15

**Authors:** Laurent Vaucher, Michael G. Funaro, Akanksha Mehta, Anna Mielnik, Alexander Bolyakov, Eric R. Prossnitz, Peter N. Schlegel, Darius A. Paduch

**Affiliations:** 1 Department of Urology and Reproductive Medicine, Weill Cornell Medical College, New York, New York, United States of America; 2 Population Council, Center for Biomedical Research, New York, New York, United States of America; 3 Department of Urology, Centre Hospitalier Universitaire Vaudois, Lausanne, Switzerland; 4 Department of Cell Biology and Physiology, University of New Mexico, Albuquerque, New Mexico, United States of America; National Cancer Institute, United States of America

## Abstract

**Purpose:**

Estradiol (E2) modulates testicular functions including steroidogenesis, but the mechanisms of E2 signaling in human testis are poorly understood. GPER-1 (GPR30), a G protein-coupled membrane receptor, mediates rapid genomic and non-genomic response to estrogens. The aim of this study was to evaluate GPER-1 expression in the testis, and its role in estradiol dependent regulation of steroidogenesis in isolated rat Leydig cells and human testis.

**Materials and Methods:**

Isolated Leydig cells (LC) from adult rats and human testicular tissue were used in this study. Expression and localization studies of GPER-1 were performed with qRT-PCR, immunofluorescence, immunohistochemistry and Western Blot. Luteinizing Hormone (LH) -stimulated, isolated LC were incubated with estradiol, G-1 (GPER-1-selective agonist), and estrogen receptor antagonist ICI 182,780. Testosterone production was measured with radioimmunoassay. LC viability after incubation with G-1 was measured using 3-(4,5-dimethylthiazol-2-yl)-5-(3-carboxymethoxyphenyl)-2-(4-sulfophenyl)-2H-tetrazolium, inner salt (MTS) assay.

**Results:**

GPER-1 mRNA is abundantly expressed in rat LC and human testis. Co-localization experiments showed high expression levels of GPER-1 protein in LC. E2-dependent activation of GPER-1 lowers testosterone production in isolated rats LCs and in human testis, with statistically and clinically significant drops in testosterone production by 20–30% as compared to estradiol-naïve LC. The exposure to G-1 does not affect viability of isolated LCs.

**Conclusions:**

Our results indicate that activation of GPER-1 lowers testosterone levels in the rat and human testis. The expression of GPER-1 in human testis, which lack ERα, makes it an exciting target for developing new agents affecting testosterone production in men.

## Introduction

Estrogens are necessary for maintaining structural and functional integrity of the male reproductive tract, [1] but little is known regarding the effects of estradiol on steroidogenesis. The cellular response to estrogens is mediated through the well-described nuclear estrogen receptors α and β (ERα, ERβ), which function as ligand-dependent transcription factors; ligand-activated estrogen receptors bind to estrogen response elements (ERE) in the genome and modulate gene expression in many tissues, including those of the male reproductive tract. Animal studies indicate that estradiol modulates the function of Leydig Cells (LC), efferent tubules, and epididymis.[2] However, the net effect of estrogens on testicular function differs between species.[3]–[5] ERα has been identified in rodent LC, but not in adult human and non-human primate LC.[6] Thus, alternative pathways of estrogen-dependent regulation must exist in human testis.[7], [8] Experimental evidence suggests that a new class of estradiol receptor, GPER-1, a 7-transmembrane-spanning G protein-coupled receptor (GPCR), mediates estrogen-dependent rapid signaling in a variety of estrogen-sensitive cells and tissues.[9], [10] Our hypothesis questioned whether GPER-1 might be important in mediating estradiol’s effects on steroidogenesis in the human testis, as it has already been proven that GPER-1 is involved in regulation of steroidogenesis in fish gonads.[11] Our aim was to reveal the patterns of GPER-1 expression in human and rat testis, and to determine if GPER-1 regulates androgen synthesis in isolated rat LC and human testis. The identification of G-1, the first synthetic agonist for GPER-1[12] allowed us to differentiate between the effects of GPER-1 and ER, as G-1 binds with high selectivity to GPER-1.

## Materials and Methods

### Chemicals

17β-estradiol (E2), ICI 182,780 and DMSO were purchased from Sigma (St. Louis, MO). Triton X, protease inhibitors and LightCycler® 480 SYBR Green I Master were purchased from Roche Molecular Biochemicals (Indianapolis, IN). GPER-1 C-terminal antibody was kindly provided by Dr. Eric Prossnitz, GPER-1 N-terminal antibody was purchased from Abcam, (Cambridge, MA), and Zenon Alexa Fluor Labeling Kit from Molecular Probes, (Eugene, OR). G-1 was purchased from Calbiochem (San Diego, CA) and ^3^H-testosterone from PerkinElmer Life Science (Boston, MA). MTS Titer Cell Proliferation assay was purchased from Promega Corporation (Madison, WI). Ovine Luteinizing Hormone was provided by the National Hormone and Pituitary Program (NIDDK, Bethesda, MD). Except for estradiol, which was diluted in 30% ethanol:70% Dimethylsulfoxide (DMSO), all ligands were solubilized in DMSO and kept as 1 mM stock solutions at −20°C.

### GPER-1 Expression in Human and Rodent Testis

#### Immunolocalization of GPER-1

The GPER-1 localization was performed in sections of adult rat testis and adult human testis using immunohistochemistry (IHC) and immunofluorescence (IF) on frozen sections. A total of 10 rats and 12 human samples were available for experiments. Anti GPER-1 N- and C-terminal primary antibodies were labeled with Zenon Alexa Fluor 594 Labeling Kit. For co-localization experiments, antibodies against vimentin (for detection of Sertoli cells) or 3-β-hydroxysteroid dehydrogenase (3β-HSD) (for LC) were labeled with Zenon Alexa Fluor 488. The nuclei were counterstained with DAPI (ProLong Gold antifade, Invitrogen). Specimens without primary antibody were used as negative controls. Image analysis was performed using ImagePro for Windows with deconvolution module, after z-stack files were obtained from each fluorescent channel using a motorized-stage microscope. For IHC, tissues were fixed in Bouin’s solution, embedded in paraffin and sectioned. HistoMouse-Maxkit-PAD (Invitrogen 87–9551) was used for detection of GPER-1 primary antibody.

#### Western blotting and Immunodetection of GPER-1

To confirm specificity of the antibody, cells were lysed in 110 µl of 50 mM Tris HCL, 150 mM NaCl, 1 mM EDTA, 1% Triton X-100, 1% SDS, a mixture of protease inhibitors and sonicated on Branson S250 Digital Sonifier. Protein concentration was determined using Invitrogen Quant-it Protein assay kit and Qubit fluorometer according to the manufacturer’s recommendations. Equal amounts of protein were resolved on a 10% SDS-polyacrylamide gel, transferred to Hybond-P PVDF transfer membrane (Amersham Biosciences), probed overnight at 4°C with anti-GPER-1 N-terminal antibody (1∶200) and anti-GPER-1 C-terminal antibody (1∶20,000). Membranes were incubated with horseradish peroxidase (HRP)-conjugated secondary antibodies and antigens detected with ECL system (Amersham Biosciences). Antibodies against β-actin were used for normalization; proteins extracted from Hec50 cells were used as positive control, and GPER-1 blocking peptide as negative control. In addition, the antibody specificity was confirmed in Dr. Prossnitz’s laboratory using GFP-GPER-1 co-localization (Image 1C).

#### Quantitative RT-PCR

To evaluate GPER-1 mRNA expression in testis, quantitative RT-PCR was performed using GPER-1 primer set (sequence: L- CCTCAACACTCACACACTCTGG, R- GATGTCTGGGCTGGTGCT). Total RNA (1 µg) was transcribed using reverse transcriptase and hexamer primers to create cDNA libraries from whole adult rat testes and isolated adult rat LC. For human studies, total RNA was extracted from testis samples obtained from patients with obstructive azoospermia with normal spermatogenesis. Expression was normalized to β-actin. LightCycler® 480 SYBR Green I Master was used to prepare master mix for qRT-PCR. Standard curves were generated for the target and housekeeping genes. Melting curves were analyzed for each reaction. All experiments were performed using a thermo-cycler (LightCycler 480, Roche) with the following settings: pre-incubation, 10 min at 95°C, amplification: 45 cycles, 95°C×10s, 60°C×10s, 72°C×12s.

### GPER-1 Regulation of Androgen Synthesis

#### Leydig cell preparation

Adult male Sprague-Dawley rats were purchased from Charles River Laboratories (Wilmington Mass). A total of 10 animals were sacrificed by CO_2_ asphyxiation according to an approved animal protocol (protocol 91200-2R, Rockefeller University). Purified LC were prepared as previously described.[13] All experiments were performed using pooled LC fractions at a time, and each experiment was performed in triplicate. Leydig cell preparations were 95% ±2 pure (3β-HSD staining). Human testis samples (50 mg) were obtained under IRB approval (protocol 0709009425) during microsurgical testicular sperm extraction. Samples from twelve patients were obtained. The tissue sample was placed in sterile PBS, subsequently suspended to assure uniform dispersion of cells, and divided into equal aliquots prior to LH stimulation and adding specific ligands.

#### Rat and human leydig cell incubation

Testosterone (T) production by LC was measured after incubation of 0.1×10^6^ cells at 34°C for 3 h in culture media.[13] For each experiment the T production by LH naïve cells and by cells stimulated with ovine LH (100 ng/ml) was measured.

#### Radioimmunoassay (RIA)

Testosterone production was measured in house by RIA in triplicate for each sample as previously described.[14] The cross reactivity of anti-testosterone antibody with estrogen was calculated at 0.52% and with ICI 182 780 at 0.16%. Cross reactivity of RIA for G-1 was less than 0.1%.

#### Dose response curves for ligands

Optimal concentrations of ligands and antagonists used in subsequent experiments (ICI 182,780 (ICI), 17β Estradiol (E2) and G-1) were determined experimentally. LC were incubated with increasing concentration of each component, and the concentration of T in the medium was measured.

#### Effect of estradiol, G-1 on testosterone production in isolated leydig cells

The isolated LC were incubated with vehicle, E2, G-1, and E2 plus G-1 together with and without LH stimulation. After three hours, the production of testosterone per 0.1×10^6^ cells (rats), or per mg of tissue (human testis) was measured in each of the vials. Experiments in humans were performed using 12 testis samples.

#### Balance between ER and GPER-1 signaling

To clarify the role of ER signaling plays on testosterone production, the isolated LC were pre-treated with the pure ER antagonist ICI 182,780 (ICI) before exposing cells to E2 or G-1. ICI 182,780 does not block the GPER-1 activation.

#### Secondary signaling through GPER-1

To evaluate if GPER-1 modulates testosterone production through mechanisms of GPCRs pathways, the isolated LC were pre-incubated with pertussis toxin (PTX), which inhibits the inhibitory G*αi* subunit of G proteins in LC. 100 ng/ml PTX was added to the medium for 15 minutes prior to adding G-1 and E2 ligands, as described previously, [15] and the T production was measured subsequently.

#### Effects of G-1 and DMSO on leydig cell viability

In order to exclude toxic effects of G-1 or vehicle (DMSO) on LC, the cell viability was measured after 3 and 12 hours of incubation with and without increasing concretions of G-1 and DMSO.

#### MTS assay

LC viability was evaluated using the MTS Cell Titer Cell Proliferation assay according to the manufacturer’s instructions.

#### Statistical analysis

Prism 5 and JMP for Macintosh were used for statistical analysis (GraphPad Software, Inc, and SAS). Statistical significance between groups was tested using t-student and ANOVA tests with post-hoc analysis as appropriate, p values of<0.05 were considered statistically significant.

### Ethics Statement

All of the work conducted within the experiments was carried out under IRB approval. Human testis samples were obtained and subsequent experiments were carried out with the approval of the Weill Cornell Medical College institutional review board (protocol 0709009425). It was decided by the IRB that neither written nor verbal consent was necessary for obtaining these human tissue specimens, as the samples obtained and used in this study were waste or to-be-discarded samples. Animal studies were carried out under the approval of the Rockefeller University IRB/IUCAC protocol 91200-2R. Per protocol, rats were sacrificed with CO_2_ prior to any further study.

## Results

### Localization and Expression of GPER-1 in Leydig Cells and Rats Testis

**Figure 1:**
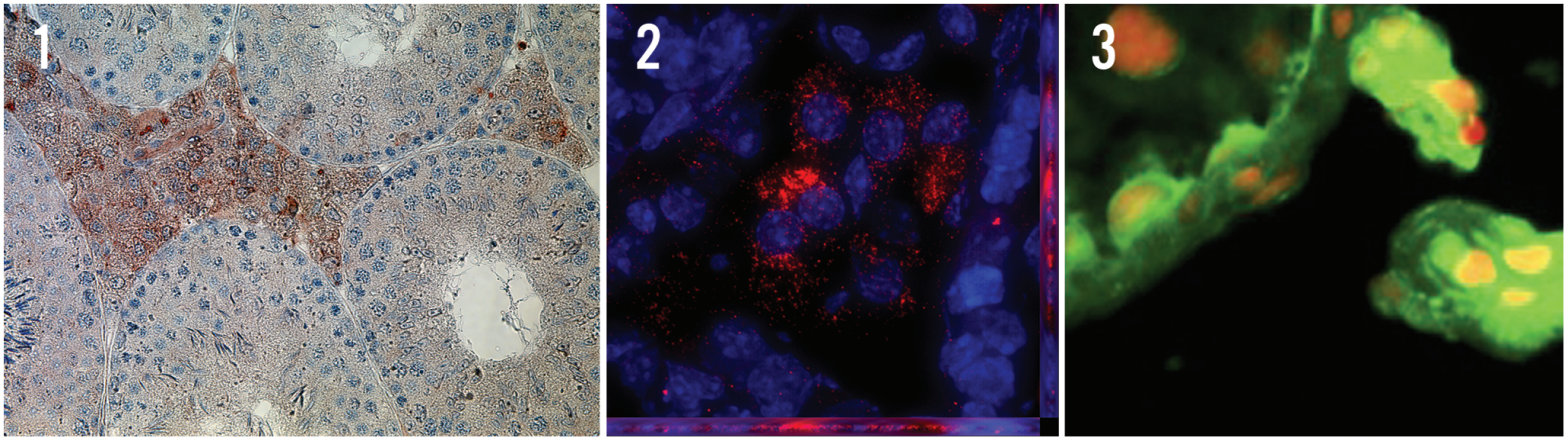
Localization studies of GPER-1 in rat testis. A. Immunohistochemistry imaging of adult rat testis. GPER-1 antibody is labeled in red. B. Immunofluorescence localization analysis of adult rat testis, with GPER-1 labeled in red, and nuclei conterstained with DAPI (blue). C. High resolution Z-stack imaging with 3D deconvolution obtained from each fluorescent channel using motorized microscope.

IHC and IF studies with antibodies against GPER-1 and 3-βHSD showed abundant expression of GPER-1 within rat and human LC. IF studies show a strong signal for GPER-1 in rat LC (Figure 1A). High-resolution Z-stack imaging with 3D deconvolution indicates that in LC GPER-1 is expressed predominantly within the cytoplasm (i.e. not the plasma membrane) and most likely within the endoplasmic reticulum, which is consistent with data found in breast cancer cell lines (Figure 1B). [16], [17].

**Figure 2:**
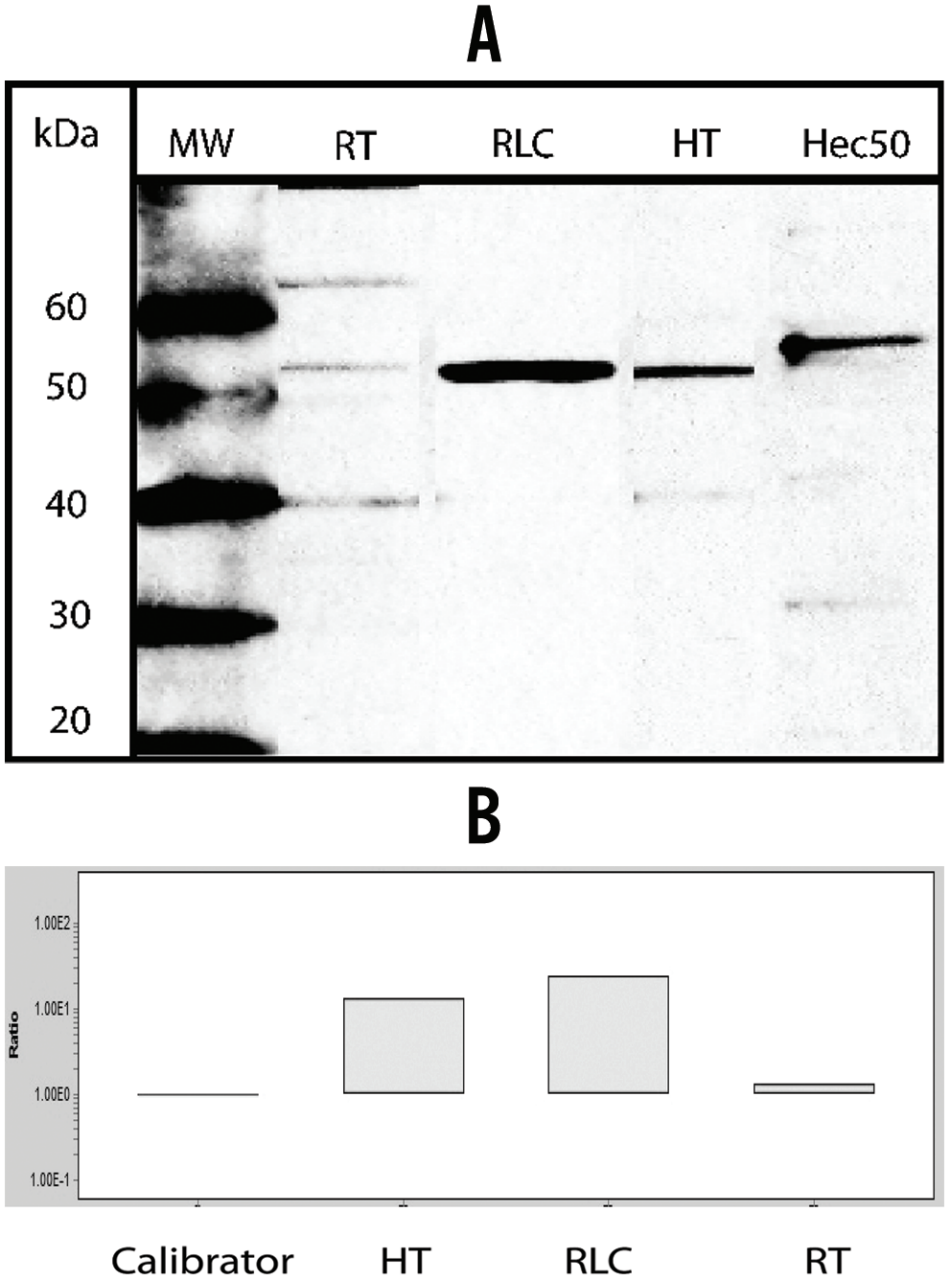
Protein/RT-PCR studies of GPER-1 in rat and human testis. A. Western blot analysis of GPER-1 expression in total protein extract from adult rat testis, isolated rat Leydig cells, human testis and Hec50 cell line cells. B. Quantitative RT-PCR in adult rat testis, isolated rat Leydig cells and human testis. RT: rat testis, RLC: Rat Leydig cells, HT: Human testis.

Western blotting using anti GPER-1 C- and N- terminal antibodies detected GPER-1 in total protein extracts from isolated rat LC, human testis and Hec50 endometrial cancer cells (Figure 2A). Although the predicted molecular weight of the GPER-1 protein is 40 kDa, GPER-1 can undergo extensive glycosylation. Experiments with blocking peptide verified that GPER-1 is expressed by the presence of strong band between 50 and 60 kDa as observed in our experiments.

Quantitative RT-PCR in human and rat showed modest expression of GPER-1 in whole testis RNA, with a significant increase (10x) in expression of GPER-1 in isolated LC which is consistent with the results of IHC showing predominant expression of GPER-1 in LC (Figure 2B).

### Dose Response Effects of G-1, 17β-estradiol and ICI 182,780 on Leydig cell Testosterone Production

**Figure 3:**
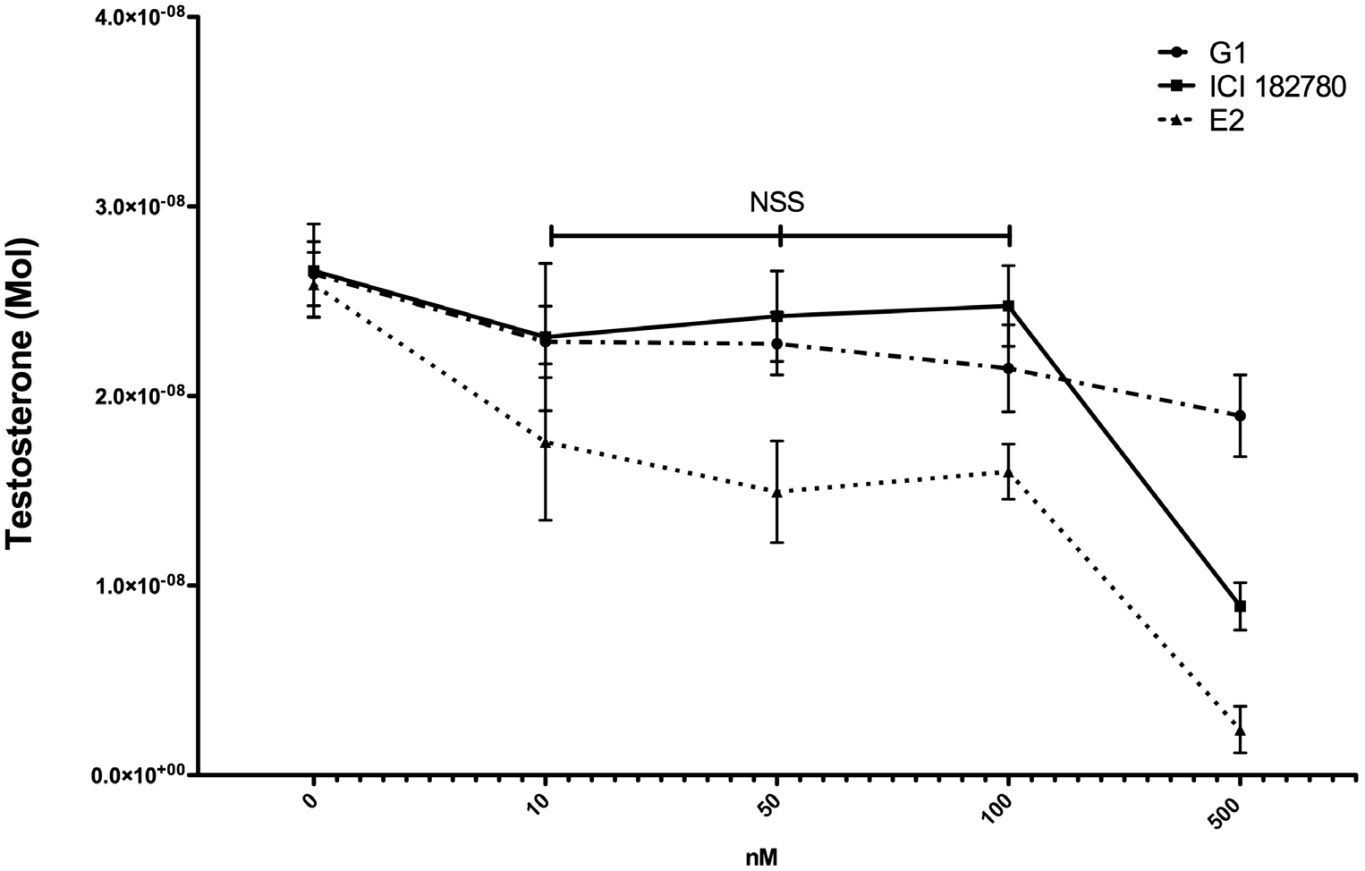
Dose response analysis of testosterone production in rat Leydig cells with E2, G-1 and ICI incubation. Final concentration of 10, 50, 100 and 500-statistically significant difference between 10, 50 and 100 nM.

Dose response analysis for E2, G-1 and ICI with final concentrations of 10, 50, 100 and 500 nM of each ligand showed that there were no statistically significant differences in testosterone production at concentration up to 100 nM (Figure 3). Concentrations of 100 nM for each ligand were used in the subsequent experiments and correspond to concentrations used by others.[18].

### Inhibition of Testosterone Production by 17β-estradiol and GPER-1 Agonist G-1

**Figure 4:**
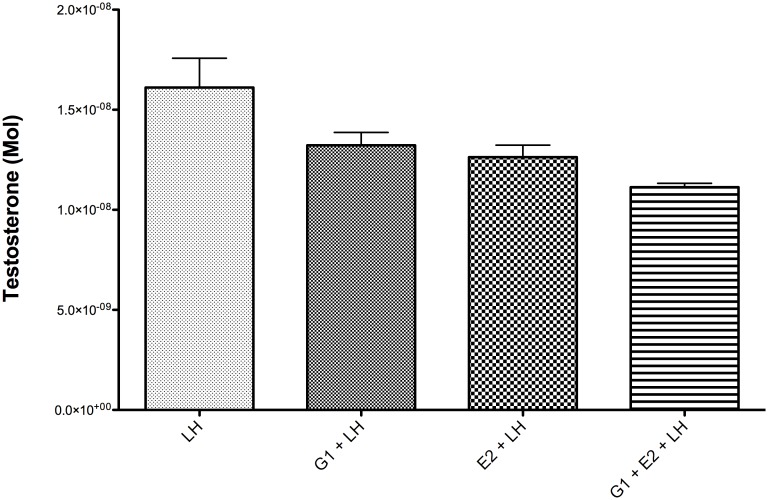
Testosterone production in isolated LH stimulated Leydig cells after 3 hours. Co incubation with G-1, E2, G-1 and E2.

In isolated LC stimulated with LH, a reproducible and statistically significant decrease in testosterone production was observed in all experiments after 3 hours of incubation with estradiol (p<0.05) (Figure 4). To answer the question if GPER-1 signaling is involved in the observed effects of estradiol on steroidogenesis, G-1, a selective agonist of GPER-1, was used. The treatment of LH-stimulated LC with G-1 showed a reproducible, statistically significant drop in testosterone production of 20% (p<0.05) These results provide supporting evidence that activation of GPER-1 signaling pathways has an inhibitory effect on testosterone production in LC. Addition of G-1 and E2 to LH stimulated cells demonstrated a inhibitory effect in LH-stimulated cells, with a 30% decrease in testosterone production, which may indicate synergistic effects of ER and GPER-1 signaling on testosterone production, or be secondary to an increased concentration of GPER-1 activating ligands, in our case G-1 and E2 to 200 nM.

### Modulation of Testosterone Production by ICI 182,780

**Figure 5:**
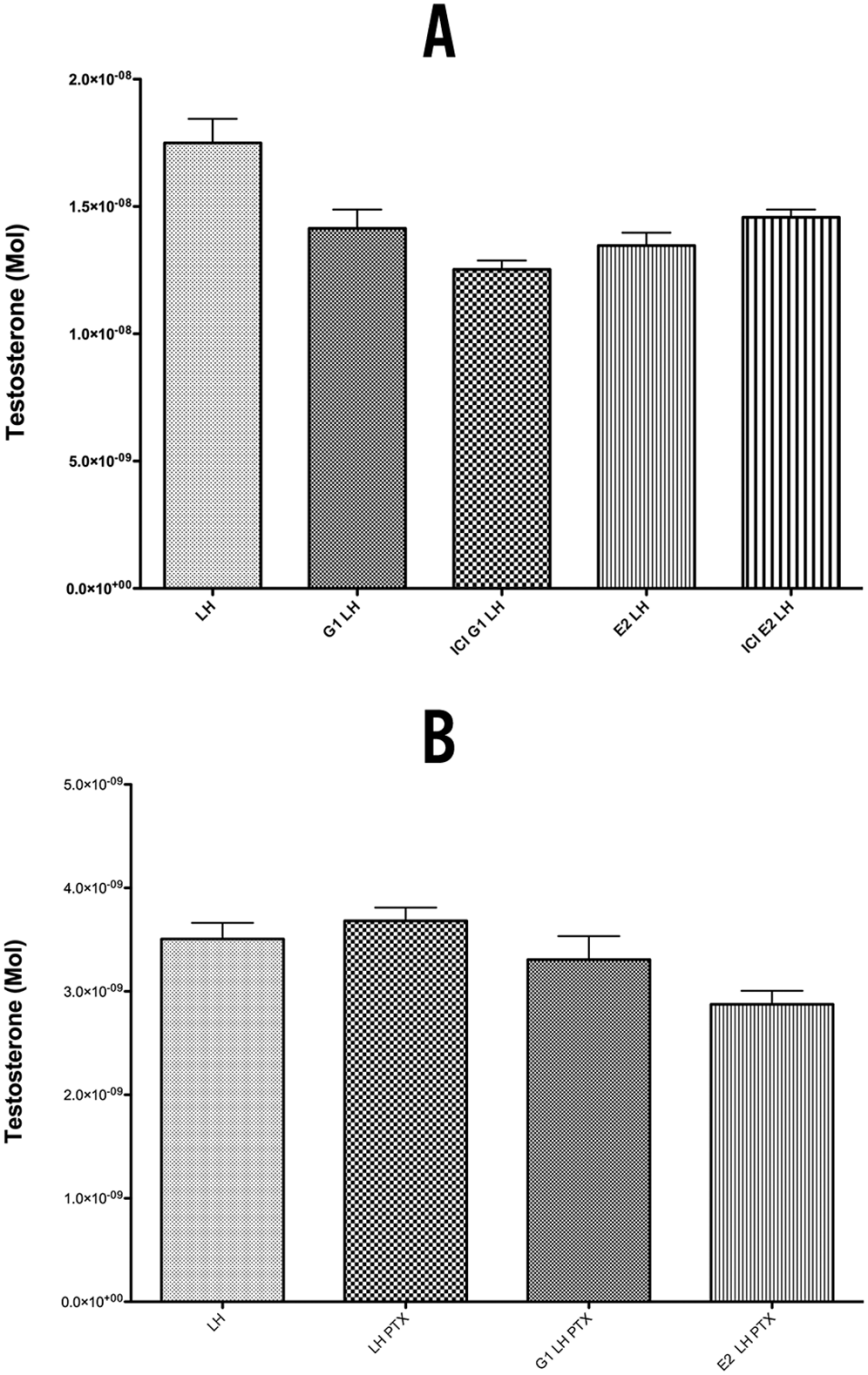
Testosterone production in isolated LH stimulated Leydig cells after 3 hours. A. Co-incubation with pure estrogen receptor antagonist ICI 182,780. B. Pre-incubated cells with pertussis toxin (PTX). 100 ng/ml PTX was added to the medium for 15 minutes prior to adding G-1 and E2 ligands.

In order to exclude modulatory effects of nuclear receptor ERα and ERβ, independently or in crosstalk with GPER-1 on steroidogenesis, we used the pure estrogen receptor antagonist ICI 182,780 (Figure 5A). Co-incubation of ICI and E2 in LH-stimulated cells showed an 8% increase in testosterone production as compared with E2 LH-stimulated cells, indicating that the classic nuclear receptor pathways through ERα and ERβ play minimal roles in the effects of estradiol on steroidogenesis. This observation was further strengthened by results from co-incubation of G-1 with ICI, which produced a 12% decrease in testosterone production as compared to G-1 alone. These data are consistent with the antagonist effect of ICI on ERα and ERβ receptors, but the agonist effect on GPER-1.[19] As human testis does not express ERα, incubation with E2, with or without ICI, did not show any statistically significant difference (data not shown).

### Effects of DMSO on Steroidogenesis

In response to concerns that vehicles like DMSO, which are used to deliver steroids and steroid receptor ligands, may themselves modulate testosterone production, [17] the cells were incubated with DMSO at the same final concentration of DMSO (0.05–0.1%) as would be used if ligands were added to the medium. There was no statistically significant difference in testosterone production in LH-stimulated cells incubated with or without DMSO.

### Effects of Different Concentrations of G-1 on Leydig Cells Viability

**Figure 6:**
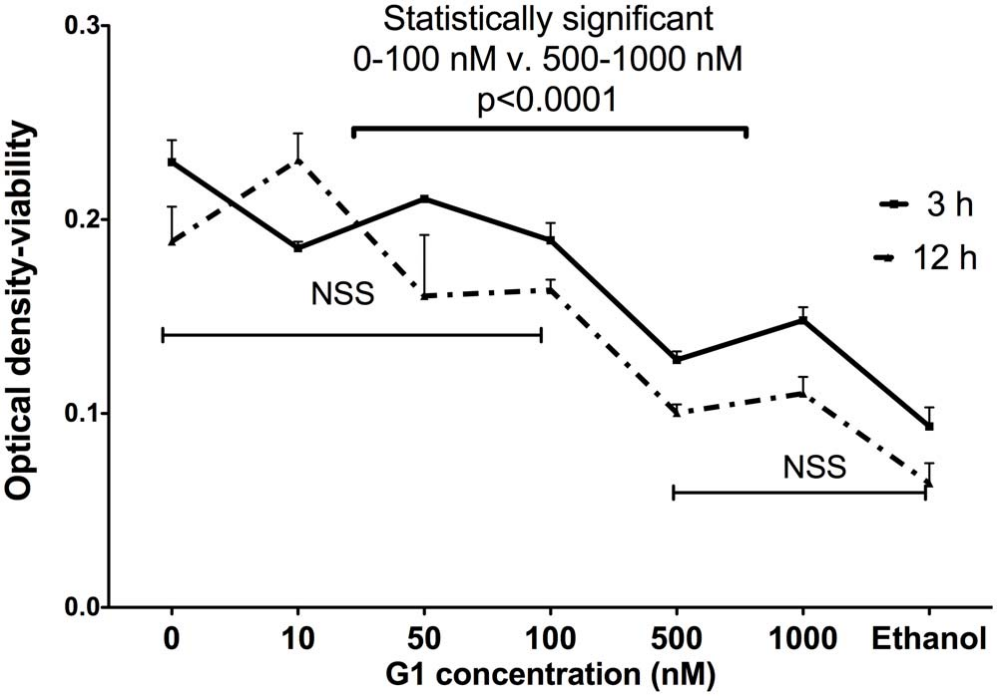
MTS viability assay performed with different concentrations of G-1, after 3 and 12 hours. Data was analyzed using two-way ANOVA with time and concentrations as factors.

To evaluate if G-1 has negative effect on cell viability and to answer the question if observed lower production of testosterone is a result of detrimental effects on LC, the MTS viability assay was performed using different concentrations of G-1. Data was analyzed using two-way ANOVA with time and concentrations as factors. The G-1 has no significant effect on Leydig cell vitality up to concentration above 100 nM; G-1 at concentration of 1000 nM decreased vitality of LC by 30% after 3 hours of incubation and 44% after 12 hours (p<0.001). G-1 at concentration of 100 nM was used in all experiments reported in our study (Figure 6).

### Inhibition of G Inhibitory Subunit of GPCR (Gαi)

To further strengthen our hypothesis that estradiol action on steroidogenesis is conducted through GPCR signaling, we used pertussis toxin, an inhibitor of the Gαi subunit. As LH also acts through a G protein-coupled receptor, we were able to observe a statistically significant increase in testosterone production between LH and LH PTX, which is in agreement with previous studies.[15], [20] Pre-incubated cells with PTX did not show the characteristic decrease in testosterone production after treatment with G-1, suggesting that blocking Gαi subunit interferes with GPER-1 signaling (Figure 5B). Moreover, a Gαi subunit might be the α subunit responsible for GPER-1 signal transduction. This observation has to be further evaluated to differentiate between effects of PTX on LH and GPER-1 signaling, since both receptors belong to the GPCR family. E2 added to cells pre-incubated with PTX led to a statistically significant, (p = 0.03) although very slight, decrease in testosterone production.

### Effect of Estradiol and G-1 on Human Leydig Cell Testosterone Production

**Figure 7:**
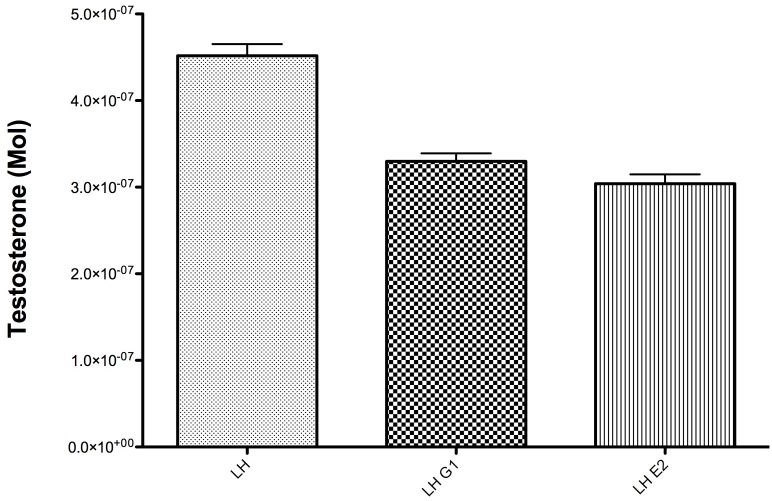
Testosterone production in human testis biopsy after 3 hours. Experiments in humans were performed using 12 testis samples. All results were pooled in statistical analysis using one-way Anova.

To investigate if GPER-1 signaling is involved in the regulation of testosterone production in human testis, testis samples were incubated with G-1 and E2 after LH stimulation. Testosterone production was first compared separately for each patient to exclude variation in steroidogenesis between subjects. However, as the percentage drop of T after G-1 and E2 was similar in all patients, all results were pooled for statistical analysis using one-way ANOVA (Figure 7). There was a statistically and clinically significant drop in testosterone production by 26% in the G-1 and E2 group after 3 hours of incubation. (p<0.05) The fact that the human testis lacks ER alpha, acting as a “natural ER alpha knockout”, and the lack of statistically significant difference between the decrease in testosterone production after incubation with G-1 or E2, provides strong evidence that GPER-1 plays a significant role in estradiol-dependent modulation of steroidogenesis in human testis.

## Discussion

Estrogen plays an essential role in the regulation of normal brain, bone, and reproductive functions in men.[21]–[23] 17β-estradiol is known to have inhibitory effects on mature Leydig cell steroidogenesis in experimental animals[24] and humans;[25], [26] Hardy and colleagues showed an inhibitory effect of estrogen on cultured rodent LC, clearly indicating that its inhibition of testosterone secretion is not solely limited to effects at the hypothalamic-pituitary-gonadal axis.[27] While evidence suggests additional modes of action, the involvement of estrogen receptors (ERα and ERβ) through direct signaling in the suppression of steroidogenesis has never been proven in human Leydig cells. Although ERβ is present in the testis, its ubiquitous nature suggests that it might not be a primary target for estrogen action.[28] In the testes, immunoexpression of ERα was described in Leydig and in peritubular myoid cells of adult rodents, [29] but not in primate or human LC [6].

GPER-1, a novel G protein-coupled estrogen receptor was shown to be involved in the effects of estrogen in normal and malignant tissue.[19] The recent availability of a specific GPER-1 agonist with negligible affinity for ERs, [12] already used by others to investigate GPER-1 activity, [30]–[32] allowed us to examine the role of GPER-1 in steroidogenesis. In the present study, we have established the expression of GPER-1 in LC, and the ability of its selective agonist, G-1, to induce a decrease in testosterone production by LC of adult rat and human testis. This decrease is dose-dependent and occurs within 3 hours, indicating most likely involvement of nonclassical, nongenomic signaling. We used the same concentrations of G-1 and E2 in our experiments, and found a similar decrease in testosterone production when we added G-1 or E2 to LH-stimulated LC. We thus conclude that the decrease in testosterone production is due to GPER-1 activation. In these short-term cell cultures, GPER-1 desensitization is unlikely to explain our results. However, as mentioned by Prossnitz, [19] G-1 may have unknown activity on other receptors or signaling proteins, and tissue-specific knockdown studies are mandatory and already planned to confirm the GPER-1 involvement in the modulation of LC testosterone production. Moreover, the lack of ERα receptor in human testis provides a unique “knock-out” model to study the effects of GPER-1 signaling, as it eliminates the classical estrogen signaling pathways. The fact that in human samples we observed exactly the same decrease in testosterone production when using estradiol and G-1 indicates that in human testis GPER-1 is an important pathway in estradiol regulation of steroidogenesis. It is also worthwhile to investigate the effects of a GPER-1 antagonist like G15 on steroidogenesis; we have performed extensive experiments and these results are forthcoming (manuscript in preparation). GPER-1 signaling is most likely the predominant mechanism of estradiol-driven downregulation of testosterone production in humans.

## Conclusion

GPER-1 signaling is a significant pathway in the regulation of steroidogenesis by estradiol in rat and human testis. This report shows that, in human and rats, detrimental effects of estrogen excess on steroidogenesis act at the testicular level through GPER-1-dependent signaling.
